# Persistent consequences of atypical early number concepts

**DOI:** 10.3389/fpsyg.2013.00486

**Published:** 2013-09-04

**Authors:** Michèle M. M. Mazzocco, Melissa M. Murphy, Ethan C. Brown, Luke Rinne, Katherine H. Herold

**Affiliations:** ^1^Institute of Child Development, University of MinnesotaMinneapolis, MN, USA; ^2^Center for Early Education and Development, University of MinnesotaSaint Paul, MN, USA; ^3^School of Education, Notre Dame of Maryland UniversityBaltimore, MD, USA; ^4^Department of Educational Psychology, University of MinnesotaMinneapolis, MN, USA; ^5^School of Education, Johns Hopkins UniversityBaltimore, MD, USA

**Keywords:** number concepts, place value concepts, whole number knowledge, number sense, dyscalculia, mathematics learning disabilities

## Abstract

How does symbolic number knowledge performance help identify young children at risk for poor mathematics achievement outcomes? In research and practice, classification of mathematics learning disability (MLD, or dyscalculia) is typically based on composite scores from broad measures of mathematics achievement. These scores do predict later math achievement levels, but do not specify the nature of math difficulties likely to emerge among students at greatest risk for long-term mathematics failure. Here we report that gaps in 2nd and 3rd graders' number knowledge predict specific types of errors made on math assessments at Grade 8. Specifically, we show that early whole number misconceptions predict slower and less accurate performance, and atypical computational errors, on Grade 8 arithmetic tests. We demonstrate that basic number misconceptions can be detected by idiosyncratic responses to number knowledge items, and that when such misconceptions are evident during primary school they persist throughout the school age years, with variable manifestation throughout development. We conclude that including specific qualitative assessments of symbolic number knowledge in primary school may provide greater specificity of the types of difficulties likely to emerge among students at risk for poor mathematics outcomes.

## Introduction

Some aspects of number knowledge involve an awareness of the meaning of somewhat arbitrary symbols (such as Arabic numerals and number words) that are used on a daily basis. This knowledge is an important predictor of later mathematics achievement (Rousselle and Noël, 2007; De Smedt et al., 2009; Krajewski and Schneider, 2009), which makes it a useful indicator of risk for mathematics learning difficulties (Mazzocco and Thompson, 2005; Jordan et al., 2007; Stock et al., 2010; Desoete et al., 2012). Although symbolic number skills begin to develop prior to schooling, they depend on instruction and typically become established in approximately first grade (Bugden and Ansari, 2011) to third grade (Girelli et al., 2000), at least for small whole numbers. Accordingly, early childhood educators' attention has been drawn to this aspect of “number sense” as a target of formal and informal learning and assessment. A challenge for educators is knowing what observable behaviors (such as counting or labeling sets) map on to important elements of number sense and how these behaviors are typically manifested in early childhood. In other words, educators may want to know what a weak number sense looks like, and which numerical behaviors reflect typical or atypical development.

One challenge in responding to this need lies in the limited delineation of number sense skills identified to date (Purpura and Lonigan, 2013), even within the subset of number skills classified as *symbolic* representations (e.g., verbal number words, written notation, physical number lines), which collectively differ from non-symbolic arrays (e.g., visual or audible sets). Measures of number sense often represent a conglomeration of numerical tasks that vary in the degree to which they overlap with each other and with non-numerical domain-general skills such as verbal memory, working memory, or spatial reasoning (e.g., Geary, 2004). Composite standardized test scores are useful for determining broad categories of mathematics difficulties, and extreme scores may also help differentiate between children with dyscalculia—a specific mathematical learning disability—and other sources of mathematics difficulties (Rubinsten and Henik, 2009). However, the dichotomous (pass/fail) nature of the item scores used to generate composites may fail to capture meaningful differences in mathematically relevant skills between individuals at a point in time when such differentiation can aid identification and instructional priorities. Indeed, broad mathematics achievement scores may underestimate the contributions of these early foundational skills (Geary et al., 2013).

In this retrospective longitudinal study, we focus on whole number knowledge in primary school as an example of foundational skills. We focus on behaviors that would be readily assessable in informal environments, and evaluate whether early indicators of atypical number concepts are associated with future computational fluency (as proposed also by Moeller et al., 2011). While recognizing that the number concepts we focus on are broad, we hypothesize that atypical errors on number knowledge tasks can meaningfully represent incomplete number concepts that persist throughout the school age years.

Accordingly, we propose that knowledge of such differences may be revealed through a qualitative analysis of responses to mathematics problems, with the goal of elucidating early number concepts that predict specific mathematics difficulties. We use this approach to assess aspects of performance failure rather than dichotomous pass/fail scoring, using frequency data from our completed longitudinal study to guide classification of typical and atypical errors that can then be evaluated as indicators of whole number concepts, and predictors of future math performance. The motivation for this approach is threefold: the aforementioned growing recognition that the number sense construct needs further delineation, the corresponding gaps in knowledge of developmental norms for fine grained numerical skills, and the high likelihood of behavioral differences in number skills given the heterogeneous nature of mathematical difficulties (Mazzocco, 2007; Rubinsten and Henik, 2009). We propose that the differences to emerge using this approach are likely to be meaningful indicators of later pervasive difficulties in specific areas of mathematics, because conceptual differences in number knowledge have been shown to persist well beyond the primary school age years (e.g., Mazzocco and Devlin, 2008; Geary et al., 2013).

A qualitative approach to assessing early number knowledge has both practical and theoretical significance. In practice, this approach is a complement to composite test scores for informal or formal assessments (e.g., differential diagnosis), and may be a more sensitive indicator of specific future mathematics outcomes. Theoretical contributions of qualitative error analyses provide for a more detailed understanding of developmental and individual differences in children's number sense and concepts. Although we do not claim that a qualitative approach is novel in research or assessment (e.g., Ginsburg, 2003), we do propose that it is an overlooked source of meaningful insights in the search for individual differences in number skills that do not necessarily conform to variation along a continuum. We use this approach to test the hypothesis that atypical errors that reflect numerical misconceptions in primary school are linked to aberrant responses observed late in middle school (Grade 8). This tests the broader notion that when early number concepts go awry, the consequences can persist.

## Materials and methods

### Participants

Participants were drawn from a longitudinal study of mathematics ability and disability described elsewhere in greater detail (Mazzocco and Myers, 2003). The initial participant pool was recruited from kindergarten classrooms in a large and socio-economically diverse public school district in the greater Baltimore, Maryland metropolitan area (which does not include schools in Baltimore city), from schools identified as having relatively low rates of mobility (to enhance retention in the longitudinal study) and low rates of free or reduced lunch participation (FRLP; as a filter for poverty). At the onset of the study, the mean FRLP rate was 16.5% (range = 1.58–29.04%) and the mean mobility rate was also 16.5% (range = 6.8–18.9%). All 445 kindergartners with proficient English were invited to participate, and 249 (120 boys) enrolled. The sample was predominately white (86%). A total of 210 participants remained in the study for at least 4 years. The sample for the present study was drawn from this group.

The present study focused on a number writing task administered during Years 03 and 04 of the longitudinal study, when most participants were in Grades 2 and 3 (except for nine of the 210 participants who had repeated a school grade). At Grade 2 the children ranged in age from 7.0 to 8.9 years (mean = 7.78, *SD* = 0.34). All 210 children were included in analyses of Grade 2 and 3 math performance (eight had repeated kindergarten or Grade 1, and one repeated Grade 2).

Some of our research questions were related to mathematics achievement status, which we determined using scores from the Test of Early Mathematics Ability—Second Edition (TEMA-2) from Kindergarten to Grade 3 (described subsequently). For those analyses, 17 children met criteria for mathematics learning disability (MLD; mean age = 7.85 years), 26 met criteria for low mathematics achievement (LA; mean age = 7.92), and 123 met criteria for typical achievement in mathematics (TA; mean age 7.74 years). The remaining 44 participants were excluded from analyses based on mathematics achievement status because their TEMA-2 scores were too inconsistent over time to confidently meet criteria for MLD, LA, or TA. Thus, 166 participants were included in the final study sample for analyses pertaining to MLD status.

Finally, for analyses focused on long-term predictors of Grade 8 performance, the sample included all 153 children who participated in the overall longitudinal study through Year 09 (mean age 13.83 years). Most of these children were in Grade 8 in Year 09 of the overall study, but eight were in Grade 7 (six had repeated kindergarten or Grade 1, one repeated Grade 6, and one repeated Grade 7).

### Materials

#### Primary school mathematics tasks

***Test of Early Mathematics Ability—2nd Edition.*** (TEMA-2; Ginsburg and Baroody, 1990). The TEMA is a standardized assessment of formal and informal mathematics knowledge normed for use with children ages 3–8 years. The TEMA-2 includes a wide range of numerical and mathematics items, such as counting aloud, counting sets, using one-to-one correspondence, number constancy, reading and writing numerals, number line concepts, and solving verbal or written arithmetic problems. Raw scores on the TEMA-2 are converted to age-referenced composites, which we used to determine participants' overall level of mathematics ability in Grades K to 3.

We used sample-based percentiles to determine mathematics ability group classification (as described elsewhere in detail, Murphy et al., 2007). Children who consistently performed below the 11th percentile on the TEMA-2 were classified as having MLD, whereas those consistently performing in the 11–25th percentile were classified as having low mathematics achievement (LA). Children consistently performing above the 25th percentile were classified as having typical achievement in mathematics (TA). Consistency was defined as criteria being met for at least half of all years in the study, and within the 95% confidence range for all years. Note that our criteria for determining MLD status classification were aligned with reported prevalence of MLD (~6–11% as reviewed by Shalev, 2007) and we relied on sample-based vs. standard normative percentiles because our use of the TEMA-2 throughout the longitudinal study (to maintain consistency after a third edition was published) led to inflated standard scores from outdated norms.

***Written Numbers Task.*** We focused on select number concept items given in the context of the TEMA-2 as potential predictors of later computational errors. Data came from the third and fourth years of the longitudinal study (Grades 2 and 3). For these items, children were asked to write the smallest, and the largest, one-, two-, and three-digit number, for a total of 6 trials per participant. Based on standardized scoring on the TEMA-2, there were two acceptable correct responses to the smallest one-digit number (0 and 1). The remaining five trials each had one acceptable correct response (9, 10, 99, 100, and 999, respectively). The criterion for passing the overall set of trials was six correct responses, and standardized scoring yielded one total dichotomous pass/fail score. In contrast, the scoring criteria for types of errors made were established as part of the present study, and applied individually to each trial such that the range of possible scores for number correct, number of errors, and number of specific error types was 0–6 (as described in more detail in the Results section). These scores from Grades 2 and 3 were used to predict performance on the Fast Math Test at Grade 8.

#### Grade 8 arithmetic fluency

***Fast Math Test*** (FMT; Mazzocco et al., 2008). The FMT is an investigator-designed, timed, paper and pencil task used to evaluate computational fluency. In this study, we used scores from the FMT as an outcome variable in analyses with Written Number task performance as the predictor. The FMT includes 8 pages, each comprised of 18 problems, with pages alternating between two levels of difficulty (4 pages of easy problems, and 4 pages of difficult problems), two operations (4 pages of addition, 4 pages of multiplication), and two sets of identical problems presented in a different order. For each operation, “easy” problems involve one- and two-digit number combinations familiar to most middle school students that are typically solved by retrieval (e.g., 7 + 7), and “hard” problems typically require “regrouping,” such that retrieval is an unlikely sole or primary strategy (e.g., 17 + 14). The FMT was administered at Grade 8 only. Test-retest reliability on this task was good. The Pearson (*r*) correlation between two *identical* pages was 0.83; correlations between mixed and grouped pairs of the same problem set ranged from 0.62 to 0.79 (Mazzocco et al., 2008).

#### Primary school and grade 8 mathematics performance associations

***Written Number and FMT performance.*** In this study, the outcome variable paired with the Written Numbers Task was drawn from the error coding of the FMT. In our previous work, we demonstrated that common and uncommon types of errors are observed on the FMT (Mazzocco et al., 2008). In the present study, we focused on uncommon place value errors that may represent a fundamental misconception about numbers, unlike more common miscalculation errors. Specifically, these place value errors involved numbers added across tens and ones places (referred to as NAATO errors in the original report), such as summing 6 + 2 and 1 + 3 when solving 16 + 23, thereby obtaining a sum of “48” or “84”). These errors were rarely observed among 8th graders completing the FMT, and we hypothesized that they would be related to the infrequent errors made on the Written Numbers Task—not simply because of their relative rarity but because both may reflect incomplete mastery of number concepts. Additional error types on the FMT are summarized in Table 3. Finally, we hypothesized that this incomplete mastery of number concepts would promote greater use of finger counting on the FMT—an infrequent strategy by 8th grade—and thus looked at the number of FMT items on which children explicitly used finger counting.

### Procedures

All children were tested individually by a female examiner. Parent consent and child consent were obtained in accordance to human subjects approved protocols. Testing sessions during Grades 2, 3, and 8 were approximately 90–120 min, divided into two sessions. These sessions occurred within 2 weeks of each other with rare exception; during Grade 8, some sessions occurred on the same day, pending participants' availability.

Most of the data were collected in school environments. In these cases, children were tested in their own school, in a quiet room occupied by only the student and examiner. The exception to this arrangement occurred when children moved to a non-participating school in a district for which we did not have in-school research testing privileges, or if a parent preferred to have the child tested in our laboratory. In these instances, testing occurred in a small quiet room occupied by only the student an examiner. Out-of-school testing occurred very infrequently in the primary grades, so the sample was too small to warrant statistical comparison. Upon request, Grade 8 assessments were conducted in a community-based environment (e.g., library meeting room) occupied by only the student and examiner. All data were scored, double scored, and entered twice independently and verified until all errors had been detected and corrected. Analyses were conducted using SPSS version 20 and R version 2.15.2.

## Results

### Analyses related to whole number knowledge

#### Developmental trends and effects of mathematics ability group on total score

We ran preliminary analyses to verify anticipated effects of grade and math achievement group (TA, LA, or MLD) on overall accuracy, using a 3 (Group) × 2 (Grade) repeated measures ANOVA on the total number of correct responses (range = 0–6). Main effects were confirmed for Grade, *F*_(1, 163)_ = 60.90, *p* < 0.0001, η^2^ = 0.272, with overall accuracy increasing from Grades 2 to 3 (Table 1); and for math achievement group, *F*_(2, 163)_ = 69.08, *p* < 0.0001, η^2^ = 0.418. Children with MLD made fewer correct responses relative to the LA or TA groups, even at Grade 3, *p*s < 0.009. The small but significant Grade × Group interaction, *F*_(2, 163)_ = 7.96, *p* = 0.001, η^2^ = 0.089, reflected larger increases in accuracy over time for the MLD group, likely due to ceiling effects.

**Table 1:** **Mean (and SD) number of correct responses out of 6 on Written Numbers Task among children with TA, LA, or MLD**.

**Participant group**	**Grade 2**	**Grade 3**
TA (*n* = 123)	5.29 (0.84)	5.76 (0.67)
LA (*n* = 26)	4.81 (1.17)	5.50 (0.71)
MLD (*n* = 17)	2.59 (1.80)	4.18 (1.78)
All groups (*N* = 166)	4.93 (1.30)	5.56 (0.97)

**Figure 1:**
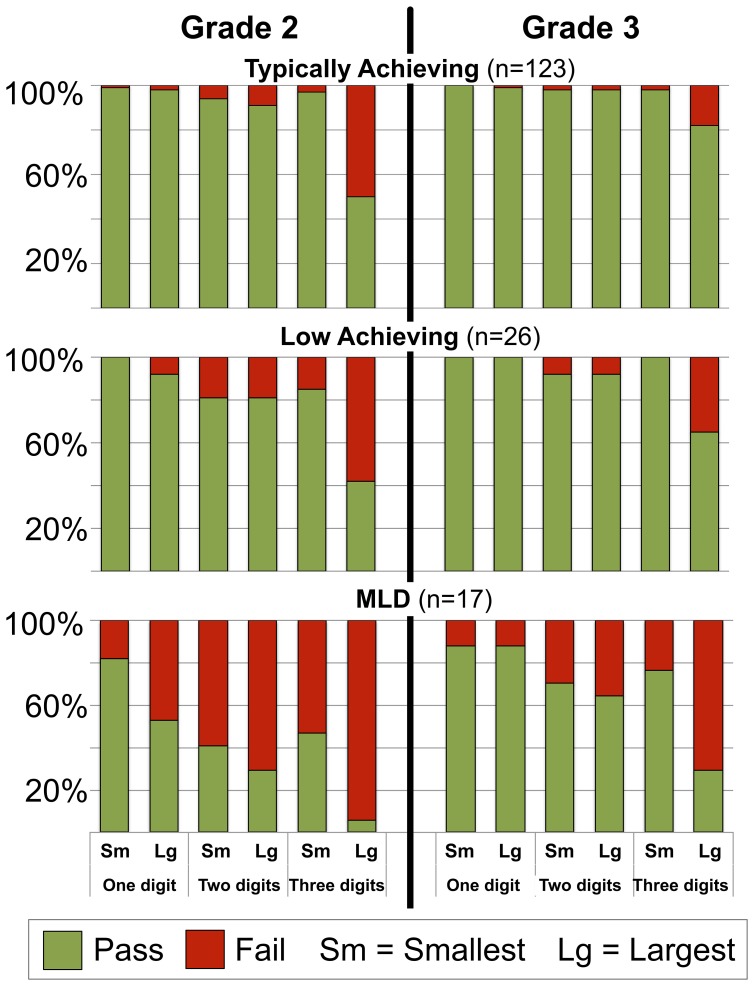
**The percentage of all children in the study who correctly or incorrectly responded to each of six items on the Written Numbers Task.** This performance summary reveals developmental and group differences from Grades 2 to 3 among children with typical achievement (TA) or low achievement (LA) in mathematics or mathematical learning disability (MLD).

These analyses of *how many* errors children made on the Written Numbers Task reveal normal developmental trends in accuracy and quantitative differences in mathematics performance across mathematics achievement groups. Figure 1 further illustrates developmental trends across easier-to-harder items (that is, one-, two-, and three-digit numbers) and the exaggeration of this effect in children with MLD. Whereas the effect of *grade* appears driven primarily by gains in knowledge of the largest three-digit number achieved between Grades 2 to 3, the main effect of *group* appears largely driven by the much larger proportion of children with MLD who do not make this shift at this time period.

Are these qualitative group differences significant? In both grades, many children with MLD failed even the 2-digit item(s), whereas most children with LA or TA did not. Very few children failed to identify the smallest one-digit number as either 1 or 0, but of the five that did fail, 4 had MLD (vs. 1 of 126 with TA, Fisher's Exact *p* < 0.01; and 0 of 26 with LA, Fisher's Exact *p* = 0.055). This pattern of performance veered from the more typical developmental pattern revealed by the data, and justified the following qualitative analyses.

#### Qualitative analyses of written numbers task errors

Does the *type* of errors made vary across math achievement group? To address this question, it was necessary to classify error types. Our *a priori* hypotheses focused on developmentally appropriate vs. idiosyncratic responses, which we operationalized in terms of *frequency* of errors made in Grade 2 across all trials and all students (210 in the study, plus 14 second graders excluded from the study due to missing data in Grade 3). Of the 1344 individual responses generated by these 224 second graders, 1068 were correct and 276 were errors. (Criteria for correct responses appear in the Methods section and in Table 2).

**Table 2:** Classification (and counts) of 224 second graders' responses showing correct responses, frequent errors, and a sample of infrequent errors^*^ on individual Written Numbers Task items.

**Response type**	**One digit**				**Two digit**				**Three digit**			
	**Smallest**		**Largest**		**Smallest**		**Largest**		**Smallest**		**Largest**	
Correct	0	(118)	9	(200)	10	(192)	99	(180)	100	(196)	999	(87)
	1	(95)										
All frequent errors		(0)		(0)	11	(9)	90	(12)		(0)	199	(20)
											900	(46)
**Infrequent errors**												
Representative infrequent errors	2	(4)	2	(2)	19	(1)	23	(1)	102	(1)	102	(1)
	4	(1)	3	(2)	22	(2)	58	(2)	104	(1)	236	(1)
	6	(1)	12	(1)	51	(1)	89	(1)	109	(2)	308	(1)
									110	(2)	590	(1)
									197	(1)	653	(1)
									532	(1)	800	(1)
									910	(1)	901	(3)
Total infrequent errors made:		(11)		(22)		(23)		(31)		(25)		(67)
“Don't know”		(0)		(2)				(1)		(2)		(3)
No response										(1)		(1)

* By definition, frequent errors were those made by >3% of all participants (≥7/224 participants), and infrequent errors were those made by fewer than 3% (<7) of all participants. Responses of “I don't know” and non-responses were considered errors but were not classified as either frequent or infrequent.

Errors were categorized as frequent or infrequent. A *frequent* error was produced by more than 3% (≥7) of all second graders in the study. There were four errors classified as frequent, which collectively occurred 87 times across trials and were made by 82 children. The mean number of children making any of the four frequent errors was 21.75 (range = 9–46). Thus, by definition, each frequent error was made by several children.

An error was categorized as *infrequent* if it was produced by fewer than 3% (<7) of all second graders in the study. Across all trials, 111 unique errors were classified as infrequent, which collectively occurred 179 times and were made by 81 children. The mean number of children making one of the 111 specific infrequent errors was 1.61, (range = 1–6). On each trial, the mean number of children who made any given infrequent error ranged from 1.32 to 2.75. Thus, by definition, infrequent errors were quite idiosyncratic in that each was made by very few children. Table 2 includes a summary of responses observed among all 224 second graders enrolled in the larger longitudinal study.

Very few responses (10 of 1334) were reports of “I don't know” (*n* = 8) or no response at all (*n* = 2), collectively made by five children (four of whom also made infrequent errors). “I don't know” responses were neither frequent nor idiosyncratic, so they were omitted from comparisons of frequent vs. infrequent responses, which led to the exclusion of data from one child whose “I don't know” error was his only error.

#### Do children with MLD make significantly more infrequent responses?

**Figure 2:**
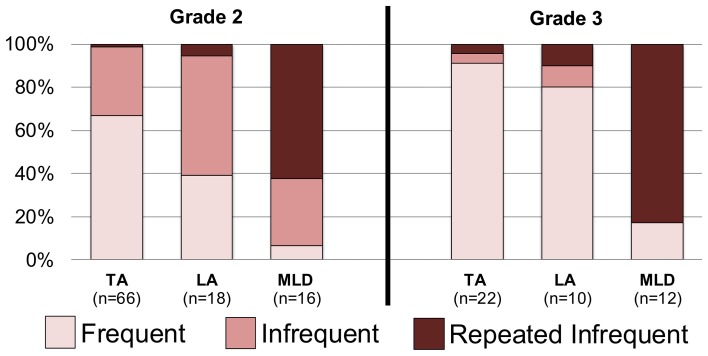
**The percentage of error types made by children who made errors on the Written Number Task at Grades 2 and/or 3, reported separately for children with typical achievement (TA) or low achievement (LA) in mathematics or mathematical learning disability (MLD).** Repeated infrequent errors indicate making an infrequent error during both years of the study, although not necessarily the same infrequent error.

We evaluated the number of infrequent errors made using a 2 (Grade) × 3 (Mathematics Achievement Group) ANOVA with only the 108 children who met criteria for MLD, LA, or TA, were tested in both Grades 2 and 3, and made at least one error in either grade (excluding the child whose only error was an “I don't know” response). There were main effects of Grade, *F*_(1, 105)_ = 34.68, *p* < 0.0001, η^2^ = 0.248, and Group, *F*_(2, 105)_ = 32.55, *p* < 0.0001, η^2^ = 0.383. The number of infrequent errors decreased from Grades 2 to 3 (from 1.45 to 0.62); across grades, children with MLD made more infrequent errors (2.25) than did children with either LA (0.52) or TA (0.32), *p*s < 0.0001 (the latter two frequencies did not differ from each other, *p* = 0.355). There was a small but significant Grade × Group interaction, *F*_(2, 105)_ = 2.78, *p* < 0.03, η^2^ = 0.068, reflected in Figure 2. Although the proportion of children making an infrequent error decreased from Grades 2 to 3, most children with MLD still did so in Grade 3. In fact, the number of infrequent errors made in Grade 3 was significantly different from zero for the MLD group only, *t*_(15)_ = 3.50, *p* < 0.01.

Perhaps the higher incidence of infrequent errors in the MLD group merely reflects greater errors of any kind among this group. If so, then their *frequent* errors should also be more prevalent. We repeated the previous 2 × 3 ANOVA, this time with the number of frequent errors as the outcome variable. Neither main effect emerged as statistically significant (*p*s > 0.15). Although the MLD group had the fewest frequent errors of any group (TA = 0.49; LA = 0.52; MLD = 0.31), these differences were not significant (*p*s > 0.3). Thus, we conclude that the performance of the MLD group is characterized by infrequent errors rather than simply more errors.

#### The validity of infrequent errors as indicators of atypical number concepts

Infrequent errors were most common among children with MLD (vs. LA or TA), but some children in each group made infrequent errors, and many of these children repeatedly made infrequent errors in Grades 2 and 3. Our subsequent analyses focused on whether children made these errors regardless of mathematics ability group.

Do infrequent errors merely reflect wild guesses? We examined metacognitive evaluation measures included in the longitudinal study test battery (Garrett et al., 2006) in which children were prompted to report if they were “sure” or “not sure” of their individual response. These prompts were administered after each trial of the Written Numbers Task. Of interest was whether children who made infrequent errors were more likely to indicate uncertainty (i.e., to state that they were “not sure” of their response), relative to children who made frequent errors, and whether this difference was limited to instances of infrequent errors.

Using data for the trial on which infrequent responses were most common (Trial 6, the “largest three-digit number”), we carried out two contingency table analyses, one per grade. We found that nearly half (48%) of the participants who made infrequent errors were confident in their incorrect responses in Grade 2 (as indicated by their report of being “sure.”), and that this rate rose to 60% among the smaller cohort that made infrequent errors in Grade 3. Although the rates were lower than the rate among respondents making frequent errors in Grade 2 (48 vs. 73%; Fisher's exact *p* = 0.016) this was not the case at Grade 3 (60 vs. 70%; *p* = 0.166), perhaps due to sample size. Nevertheless, the findings demonstrate that infrequent errors were not always merely guesses, especially in third grade, when children making these errors were more likely to be certain of their response than uncertain.

### The long-term significance of error type on the written numbers task

The idiosyncratic nature of infrequent errors is perplexing, but is it meaningful? Our final set of analyses focused on whether infrequent errors in primary school are associated with computational performance at the end of Grade 8 as measured by the Fast Math Test (FMT).

First, we hypothesized that children making an infrequent error on the Written Numbers Task in Grade 3 would be more likely to make an infrequent type of place-value error on the FMT (compared to children who made either no errors or only frequent errors at Grade 3), and that this pattern would not emerge for computational errors commonly made by 8th graders. This hypothesis was supported. We found that 35% of children who made an infrequent error at Grade 3 made atypical place value errors in Grade 8, which was significantly higher than the rate of 3% among those who had not made an infrequent error in Grade 3 (odds ratio = 17.26; *p* < 0.001). Whereas 35% of children who made an infrequent error on the Grade 3 task also made a common calculation error on the FMT (tens place addition error, defined in Table 3) in Grade 8, this rate did not differ from the rate of 42% among children who had not made an infrequent error at Grade 3 (odds ratio = 0.76; *p* > 0.7). The specificity is illustrated further in Table 4, where we also report the mean number of FMT errors and *t*-test results between children who did make infrequent errors at Grade 3 and those who did not.

**Table 3:** **Types of errors coded on the Fast Math Test (FMT)**.

**Operation**	**Error type**	**Definition**	**Example**
Addition	±1	Sum is off by one (miscalculated ones place)	7 + 12 = 18
	±10	Sum is off by an order of ten (miscalculated tens place)	20 + 25 = 55
	Place value error	Adding numbers across tens and ones places (in the example, 7 + 2 = 9, and 1 + 3 = 4)	17 + 23 = 94
			17 + 23 = 49
Multiplication	Operand	A problem is solved using the wrong operand	5 × 7 = 30
	Table	The given answer is a product on the 10 × 10 multiplication table, but not the correct product	8 × 8 = 63
	Non-table	The given answer is not a product on the 10 × 10 multiplication table	7 × 3 = 26
	Regrouping	Incomplete or incorrect regrouping	10 × 8 = 108
Either operation	Operation	A problem is solved using the wrong operation	4 + 3 = 12
			4 × 3 = 7

Adapted from a table appearing in Mazzocco et al. (2008).

**Table 4:** **Mean (and SD) number of errors made on 8th grade timed calculation on the Fast Math Test (FMT), as a function of types of calculation errors and whether infrequent errors were made on the Written Numbers Task at Grade 2 or Grade 3**.

	**Infrequent errors made in Grade 2?**			**Infrequent errors made in Grade 3?**		
	**No (*n* = 102)**	**Yes (*n* = 51)**	***p***	**No (*n* = 136)**	**Yes (*n* = 17)**	***p***
**MULTIPLICATION**						
**Operand**	1.26 (1.49)	1.92 (2.12)	0.052^†^	**1.35 (1.63)**	**2.59 (2.29)**	**0.005**
Table	0.55 (0.97)	0.86 (1.40)	0.108	0.58 (1.04)	1.24 (1.68)	0.134^†^
Non-Table	0.79 (1.58)	0.86 (1.43)	0.794	0.76 (1.55)	1.29 (1.31)	0.173
Regrouping	0.61 (0.99)	0.51 (0.73)	0.531	0.54 (0.92)	0.82 (0.81)	0.233
Total	3.40 (3.24)	4.51 (4.32)	0.110^†^	3.41 (3.34)	6.65 (4.83)	0.000
**ADDITION**						
±1	0.79 (1.10)	1.16 (1.35)	0.077	0.86 (1.19)	1.35 (1.22)	0.110
±10	0.62 (0.95)	0.84 (1.36)	0.293^†^	0.68 (1.05)	0.82 (1.51)	0.607
**Place value**	**0.03 (0.17)**	**0.22 (0.61)**	**0.037^†^**	**0.03 (0.17)**	**0.59 (0.94)**	**0.026^†^**
Total	2.75 (2.25)	4.12 (4.51)	0.044^†^	2.92 (2.62)	5.47 (5.93)	0.098^†^
**EITHER OPERATION**						
Operation	0.39 (0.88)	0.65 (0.89)	0.095	0.40 (0.80)	1.06 (1.30)	0.058^†^
**RT on easy sets (seconds)**	**128.9 (31.6)**	**153.0 (43.9)**	**0.001^†^**	**131.8 (33.8)**	**177.9 (43.6)**	**0.000**

† Reflects Satterthwaite approximation for degrees of freedom to correct for unequal variances; used only in cases of unequal variance.

Second, we had hypothesized that incomplete number concepts may promote finger counting during addition, and examined the number of items on which children used this strategy on the FMT. Children who committed an infrequent error in Grade 2 were more likely to use finger counting on addition problems in Grade 8 (odds ratio = 2.81; *p* = 0.023) than were those who did not make infrequent errors at Grade 2. The odds ratio based on Grade 3 data was in the same direction, but was not statistically significant (odds ratio = 2.01; *p* = 0.211, perhaps due to small sample size). These findings do not indicate causal pathways, but they do support the notion that early number concept errors have long-term consequences.

In summary, the results on the FMT analyses indicate that, relative to children who do not make infrequent errors on the Written Numbers Task, children who make infrequent errors on this task in Grades 2 or 3 are not only slower on mathematics computations in Grade 8, but also make more errors on addition and multiplication computations in Grade 8, and this higher rate of error appears to be selective.

## Discussion

In this retrospective study based on secondary analyses, we focused on qualitative aspects of children's early number concepts. The motivation for this study stemmed, in part, from our observations over time of the persistent difficulties some children in our longitudinal study displayed, on relatively basic arithmetic skills, from kindergarten through Grade 8. The findings show how early (and easily assessed) indicators of number skills predict later performance, thereby validating those skills as potential screening points. These findings also illustrate the value of evaluating MLD based upon specific skills rather than composite performance (e.g., Butterworth, 2005), and the implications of individual differences for persistent mathematics difficulties. Specifically, we show that the occurrence and nature of atypical number concepts at Grade 3 are associated with accuracy and types of errors made on mental calculations at Grade 8. Finally, our findings support claims of qualitative differences in early number skills between children with vs. without MLD (e.g., Mazzocco et al., 2011), although claims counter to this notion have also been supported (e.g., Landerl and Kölle, 2009). We believe these findings have practical and theoretical value, despite their preliminary nature.

We examined the “smallest” and “largest” numbers children generated in second and third grade, a time by which we would expect children's single digit whole number knowledge to be well-mastered, their associations between symbolic numbers and meanings to be automatized (e.g., Girelli et al., 2000), and their familiarity with numbers to apply to 2- and 3-digit numbers. Several interesting patterns emerged from this study related to developmental and individual differences in performance accuracy on this task and to the implications of these differences for future computational fluency. First, we found anticipated developmental trends in how accurately children generated two- and three-digit numbers from Grades 2 to 3. The pattern of heightened accuracy in two- and, later, three-digit numbers parallels findings that two-digit processing is not automatically generalized to three-digit number processing (Mann et al., 2012). Next, and as anticipated, these developmental differences seen in overall accuracy were somewhat exaggerated in children with low achievement, and remarkably exaggerated among children with MLD, who continued to make errors at a much higher rate through Grade 3 (Figure 1 depicts both the developmental trends and the group differences). More importantly, interesting individual differences were observed in the nature of children's errors when generating two- and three-digit numbers.

Not all errors on the Written Numbers Task were equivalent. Some implicated developmental trends that have not been reported previously, but which align with recent evidence of a hundreds-place focus when processing three-digit numbers, at least among children in this age group (Mann et al., 2012). For instance, the most frequent *incorrect* response to prompts for the largest three-digit number was “900,” which was reported by 46 of the 224 s. graders. The number “90” was the only frequent error to prompts for the largest two-digit number (although made by only 12 s graders). Frequent errors were less common in children with MLD, despite the fact that children with MLD made more errors overall. Only one child with MLD reported that “900” was the largest three-digit number, and yet (as seen in Figure 1), most children with MLD made errors on this trial. Although only a small group of children repeatedly made idiosyncratic errors (that is, in both grades), this was a characteristic of most of the children with MLD (Figure 2). Note, however, that children rarely reported the same idiosyncratic answer in both years.

Beyond the mere emergence of these idiosyncratic errors, of particular interest is the finding that such errors observed at Grade 3 were associated with specific and unusual errors on mathematics computation 5 years later. On the one hand, these results support the notion that idiosyncratic number concepts in early childhood are a meaningful reflection of persistent number concept anomalies which may affect the foundation for later arithmetic computation (as proposed by Mann et al., 2012). On the other hand, our interpretation is far from definitive given the sample size and other limitations associated with our retrospective analysis of longitudinal data collected for a prospective study.

It is possible that the primary predictor variable that we explored here—infrequent errors in written whole numbers—is simply a repackaging of the MLD criteria used in the study. Based on these criteria, children with MLD make more infrequent errors than their primary school peers on the Written Numbers Task, they make more place value errors than their 8th grade peers (Mazzocco et al., 2008), and they are generally less accurate at evaluating their own math performance and thus produce ratings of confidence poorly calibrated with their performance (Garrett et al., 2006). Yet infrequent errors were also made by some (albeit, very few) children with LA or TA, and a few children with MLD did not make any infrequent errors. Additional support that MLD and infrequent error criteria are distinct (even if overlapping) predictors comes from the finding that infrequent errors at Grade 3 did not predict all types of Fast Math Test errors that occur with greater frequency among children with MLD.

## Conclusions and future directions

In this retrospective study, we demonstrate how a qualitative error analysis of early symbolic number knowledge reveals potential sources of individual differences that may affect mathematics outcomes 6 years later. This means that misguided early number concepts may have long-term implications. Our goal was not to definitively identify core deficits of dyscalculia or MLD, but rather to illustrate the contribution of qualitative analysis to delineating meaningful aspects of number sense by focusing on one representation of number concepts.

Although we focused on qualitative analysis of errors, *correct* responses may also be revealing. For instance, most second graders (118 of 224) reported that the smallest 1 digit number was “0,” and fewer than half (95) reported this value was “1.” In contrast, among children with MLD who correctly answered this item, most responded “1” rather than “0.” Both answers are scored as correct, but each may represent different levels of understanding of these small numbers. At issue is how well responses such as these reflect the nature of children's fundamental number concepts.

Such qualitative analyses of responses, including errors, must be evaluated relative to developmental norms. For instance, over time, errors that were considered frequent vs. infrequent must be re-evaluated. In our longitudinal study, we continued to administer the Written Numbers Task to children who failed any of the six trials in a given year, so we were able to discover that of the seven children who continued to err on trial 6 during Grade 5, all answered “900.” [Some children continued to err on this item in Grade 6 (*n* = 6), Grade 7 (*n* = 3), and even Grade 8 (*n* = 1)]. Whereas at Grade 3 this response was categorized as a *frequent* error, it became *infrequent* by Grade 5.

Our observations underscore recommendations for the use of thoughtful questioning in mathematics assessments and teaching (Ginsburg, 2003) and when seeking to differentiate delayed vs. deficient mathematics skills (Rubinsten and Henik, 2009), especially when compensatory mechanisms mask otherwise aberrant numerical processing (Murphy and Mazzocco, 2008). Teachers can remain attentive for atypical errors on an informal basis as a source of information used to guide their online and systematic decision-making about students' individual learning needs or difficulties. Information about *where* trouble may occur down the line increases the specificity of targeted interventions. Since early basic number knowledge deficits can persist throughout the school age years, we must be mindful that their manifestation varies with development, and that the inclusion of specific qualitative assessments of symbolic number knowledge in primary school can provide greater specificity of the types of difficulties likely to emerge among students at risk for poor mathematics outcomes.

## Author contributions

Michèle M. M. Mazzocco conceived and designed the study; Michèle M. M. Mazzocco and Melissa M. Murphy carried out the study; Michèle M. M. Mazzocco and Ethan C. Brown analyzed data; Michèle M. M. Mazzocco, Melissa M. Murphy, and Ethan C. Brown wrote the paper. All authors contributed to editing and reviewing the paper.

### Conflict of interest statement

The authors declare that the research was conducted in the absence of any commercial or financial relationships that could be construed as a potential conflict of interest.
